# Circular RNAs: Biogenesis, Function, and a Role as Possible Cancer Biomarkers

**DOI:** 10.1155/2017/6218353

**Published:** 2017-12-04

**Authors:** Luka Bolha, Metka Ravnik-Glavač, Damjan Glavač

**Affiliations:** ^1^Department of Molecular Genetics, Institute of Pathology, Faculty of Medicine, University of Ljubljana, Ljubljana, Slovenia; ^2^Institute of Biochemistry, Faculty of Medicine, University of Ljubljana, Ljubljana, Slovenia

## Abstract

Circular RNAs (circRNAs) are a class of noncoding RNAs (ncRNAs) that form covalently closed continuous loop structures, lacking the terminal 5′ and 3′ ends. CircRNAs are generated in the process of back-splicing and can originate from different genomic regions. Their unique circular structure makes circRNAs more stable than linear RNAs. In addition, they also display insensitivity to ribonuclease activity. Generally, circRNAs function as microRNA (miRNA) sponges and have a regulatory role in transcription and translation. They may be also translated in a cap-independent manner *in vivo*, to generate specific proteins. In the last decade, next-generation sequencing techniques, especially RNA-seq, have revealed great abundance and also dysregulation of many circRNAs in various diseases, suggesting their involvement in disease development and progression. Regarding their high stability and relatively specific differential expression patterns in tissues and extracellular environment (e.g., body fluids), they are regarded as promising novel biomarkers in cancer. Therefore, we focus this review on describing circRNA biogenesis, function, and involvement in human cancer development and address the potential of circRNAs to be effectively used as novel cancer diagnostic and prognostic biomarkers.

## 1. Introduction

Noncoding RNAs (ncRNAs) represent a large, complex, and heterogeneous group of RNA molecules that can be classified into two major classes, according to the size of their transcripts: the small ncRNAs (<200 bp) and the long ncRNAs (lncRNAs) (>200 bp) [1, 2]. The small ncRNAs include microRNAs (miRNAs), small nuclear RNAs (snRNAs), PIWI-interacting RNAs (piRNAs), small interfering RNAs (siRNAs), small nucleolar RNAs (snoRNAs), and others [1], whereas lncRNAs comprise long intergenic ncRNAs (lincRNAs), intronic ncRNAs, macroRNAs, sense ncRNA, antisense RNAs, and others [2]. In addition, circular RNAs (circRNAs) have been recently identified as a relatively large class of ncRNAs, which are widespread and abundant in a variety of eukaryotic organisms and involved in multiple biological processes [3, 4]. CircRNAs may vary in length significantly, most of them being longer than 200 nt. However, some exonic and intronic circRNAs were shown to be shorter than 200 or even 100 nt [5].

CircRNAs form covalently closed continuous loop structures, without terminal 5′ caps and 3′ polyadenylated tails. They are generated by alternative splicing of pre-mRNA transcripts, where an upstream splice acceptor is joined to a downstream splice donor, in the process of back-splicing [6–8]. In the last decade, RNA-seq and other next-generation sequencing techniques have enabled a significant breakthrough in circRNA discovery, leading to the identification and characterization of a large number of circRNAs in humans and other eukaryotes [9, 10]. Several research groups have demonstrated a conservation of circRNA expression across mammals. In addition, circRNAs appear to be stably expressed in a cell/tissue-dependent and developmental stage-specific manner [3, 9, 11, 12]. Emerging evidence reveals the importance of circRNA involvement in regulating gene expression at transcriptional and posttranscriptional levels, and, furthermore, dysregulation in circRNA expression correlates with irregularities in developmental processes and various disease states, including cancer [13–16]. Regarding the observed correlation between altered circRNA expression profiles and a cancer patient's clinical characteristics and circRNA's structural features that enable their abundance and stability in various biological samples, we focus this review on describing circRNA involvement in cancer development. In addition, we address the potential of circRNAs to be effectively used as novel biomarkers in cancer.

## 2. Biogenesis of CircRNAs

**Figure 1:**
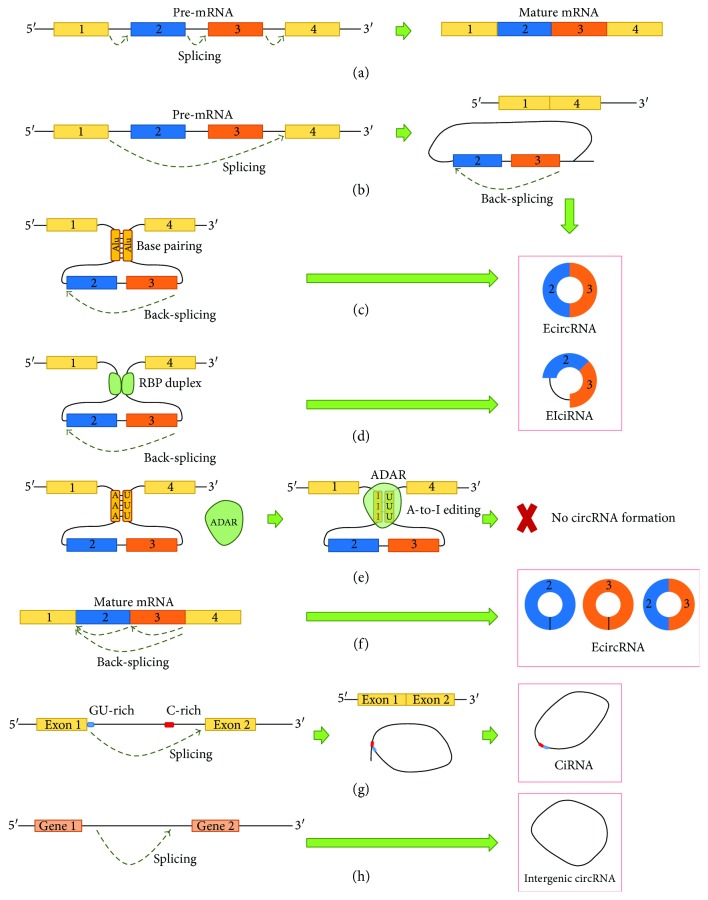
Schematic representation of circRNA biogenesis. (a) Canonical pre-mRNA splicing, yielding a mature mRNA molecule. (b) Lariat-driven circularization (exon skipping). Following canonical splicing, exons in exon-containing lariats undergo back-splicing and circularization, which results in the formation of ecircRNA or EIciRNA molecules. (c) Intron pairing-driven circularization, utilizing coupling of flanking introns by direct base pairing between *cis*-acting regulatory elements that contain reverse complementary sequences (e.g., Alu repeats). Intron pairing is followed by back-splicing and exon circularization. (d) CircRNA biogenesis, mediated by *trans*-acting factors, such as RNA-binding proteins (RBPs) (e.g., QKI, MBL/MBNL1) that bind to specific sequence motifs of flanking introns on linear pre-mRNA, dimerize, and facilitate back-splicing and exon circularization. (e) Regulation of circRNA biogenesis by the RNA-editing enzyme ADAR. ADAR destabilizes intron base pairing interactions through the action of adenosine-to-inosine (A-to-I) editing, which impairs pre-mRNA looping and diminishes exon circularization. (f) Resplicing-driven circularization. EcircRNAs may be formed from mature mRNA exons that undergo back-splicing and circularization. (g) Formation of ciRNAs from intron lariats that escape the usual intron debranching and degradation, following the canonical pre-mRNA splicing. (h) Formation of intergenic circRNAs. This figure is adapted from Wang et al. [151].

The majority of circRNAs originate from exons of protein-coding genes, frequently consisting of 1–5 exons [9]. However, they may be also formed from intronic, noncoding, antisense, 3′ UTR, 5′ UTR, or intergenic genomic regions [9, 17]. CircRNAs are generated by a spliceosome-mediated pre-mRNA back-splicing which connects a downstream splice donor site (5′ splice site) to an upstream acceptor splice site (3′ splice site) [18]. Similar to canonical (linear) splicing, back-splicing appears to be extensively regulated by canonical *cis*-acting splicing regulatory elements and *trans*-acting splicing factors. However, the regulation process of back-splicing in controlling circRNA production fundamentally differs from that of linear splicing, where the same combinations of splicing regulatory elements and factors have distinct or even opposite activity [18]. In addition, a single gene locus can produce various circRNAs through alternative back-splice site selection, when compared to canonical splicing of linear RNAs [19]. Generally, circRNAs can be generated by canonical and noncanonical splicing [20]. Regarding their biogenesis from different genomic regions, circRNAs can be categorized into four types, as determined by RNA-seq: exonic circRNAs (ecircRNAs) [9, 11, 12], circular intronic RNAs (ciRNAs) [17], retained-intron or exon-intron circRNAs (EIciRNAs) [11, 21], and intergenic circRNAs [9]. Schematic representation of ecircRNA, ciRNA, EIciRNA, and intergenic circRNA biogenesis is shown in Figure 1.

EcircRNAs are the most abundant circRNA type, accounting for over 80% of identified circRNAs, and are predominantly located in the cytoplasm [3, 9, 12], though the exact process of nuclear export remains to be elucidated. It has been suggested that ecircRNAs may escape from the nucleus during mitosis [22]. Although the exact mechanism of circRNAs biogenesis remains unclear, three models of circRNA formation have been proposed, including lariat-driven circularization (exon skipping) [12] (Figure 1(b)), intron pairing-driven circularization [12] (Figure 1(c)), and resplicing-driven circularization [23] (Figure 1(f)). It has been demonstrated that exon circularization depends on several genomic features, essential for promoting circularization. In general, exons comprising circRNAs are longer than average exons, which is especially notable for single-exon circRNAs, being approximately 3-fold longer, when compared to other expressed exons [3, 12]. In addition, several transcriptome analyses indicated a significant correlation between the presence of flanking intronic regions, containing the reverse complementary sequences (e.g., Alu elements) that may promote intron pairing, and exon circularization [12, 24, 25]. Normally, flanking introns containing inverted tandem repeats, involved in back-splicing and circRNA production, tend to be longer than introns generally, but some can be shorter than average [11]. It has also been demonstrated that relatively short (30–40 nt) inverted repeats are sufficient for intron base pairing and subsequent circRNA formation [26]. However, not all intronic tandem repeats can support exon circularization. In some cases, increased stability of intron base pairing prevented circRNA formation [26]. During the biogenesis process of circRNAs, introns may not be spliced out completely but are retained between the encircled exons in the newly generated circRNA. This phenomenon results in the formation of EIciRNAs [21]. The important role of RNA-binding proteins (RBPs) has been demonstrated in the regulation of circRNA production, which can act as *trans*-acting activators or inhibitors of the circRNA formation mechanism. Quaking (QKI) and muscleblind (MBL/MBNL1) proteins can bind to specific sequence motifs of flanking introns on linear pre-mRNA sequences, thus linking the two flanking introns together, promoting cycling and subsequent circRNA generation [27, 28] (Figure 1(d)). The process is similar to an intron pairing-driven circularization model, only that here, RBPs, after binding to specific putative binding sites, dimerize which leads to pre-mRNA looping. Conversely, the RNA-editing enzyme adenosine deaminase acting on RNA (ADAR) antagonizes circRNA production by direct binding and weakening RNA duplexes (e.g., inverted Alu repeats), through the action of adenosine-to-inosine (A-to-I) editing [25] (Figure 1(e)). High ADAR expression destabilizes intron base pairing interactions, impairing pre-mRNA looping and decreasing the likelihood of pre-mRNA circularization and circRNA formation, for a subset of circRNAs [25, 29].

CiRNA formation differs from that of ecircRNA and EIciRNA (Figure 1(g)). Stable ciRNAs can be formed, when intron lariats escape the usual intron debranching and subsequent degradation, following the canonical spliceosome-mediated pre-mRNA splicing [17]. CiRNA biogenesis depends mainly on a 7 nt GU-rich element near the 5′ splice site and an 11 nt C-rich element near the branch point site. During back-splicing, the two elements bind into a lariat-like intermediate, containing the excised exons and introns, and are cut out by the spliceosome [17, 30]. Then, generated stable lariats undergo 3′ tail degradation, which results in the formation of the final ciRNA molecule [17]. Generally, ciRNAs may be sensitive to RNA debranching enzymes and can be distinguished from ecircRNAs by the presence of a 2′–5′ junction, a residue of the lariat structure, which is evidently absent in ecircRNAs [17]. CiRNAs, along with EIciRNAs, are predominantly located in the nucleus and are believed to be involved in regulating expression of local genes in *cis* [17, 21]. In addition, sequence analyses have shown a weak but significant enrichment of conserved nucleotides between few ciRNAs and intergenic circRNAs [9]. However, there is currently very little information on the overall characteristics and biogenesis processes of intergenic circRNAs.

Despite a good deal of information on circRNA biogenesis, relatively little is known about the metabolic processing of these molecules within cells. Since circRNAs are abundant and highly stable and show resistance to exonucleases (e.g., RNase R) [9, 31], they may accumulate in the cell with a possible toxic effect. A study employing three cell lines has shown that the aggregated excessive circRNAs could be effectively eliminated from cells via released vesicles such as exosomes and microvesicles [32]. An additional study has also demonstrated similar results, where excessive circRNAs were enriched over their linear isoforms within extracellular vesicles, when compared to the producing cells [33].

## 3. Function of CircRNAs

**Figure 2:**
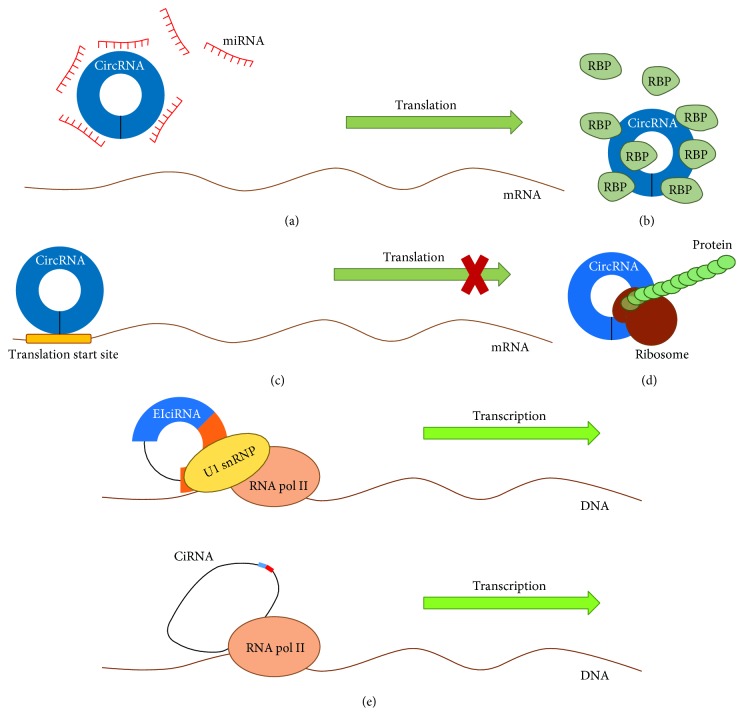
Schematic representation of circRNA functions. (a) CircRNAs may act as miRNA sponges by competing for miRNA binding sites, diminishing the effect of miRNA-mediated regulatory activities. (b) CircRNAs may act as protein sponges, by binding RNA-binding proteins (RBPs). (c) Some circRNAs may regulate protein expression by sequestering mRNA translation start sites. (d) CircRNAs may be translated to form functional proteins. (e) CircRNAs (e.g., EIciRNAs and ciRNAs) may interact with transcription complexes and enhance the expression of their parental genes.

CircRNAs have important functions in regulating gene expression and may act as miRNA sponges, RBP sponges, and regulators of transcription and translation [21, 34, 35]. Also, several circRNAs have shown the ability to be translated into peptides [18, 36–39]. Schematic representation of these circRNA functions is shown in Figure 2.

miRNAs are approximately 22 nt long ncRNAs, involved in posttranscriptional regulation of gene expression, which act by direct binding to specific target sites within UTRs of mRNAs [40, 41]. The result of such miRNA-mediated mRNA targeting is either blockage of the translation process or complete degradation of the bound mRNA molecule [41]. CircRNAs can act like miRNA sponges by competing for miRNA binding sites (Figure 2(a)), thus diminishing the effect of miRNA-mediated regulatory activities (e.g., posttranscriptional repression) [9, 34]. Indeed, it has been demonstrated that overexpression of miRNA sponge-acting circRNAs increases the expression of miRNA targets, whereas knockdown of these circRNAs had the opposite effect [34]. The human cerebellar degeneration-related protein 1 antisense (CDR1as) circRNA, also known as a circular RNA sponge for miR-7 (ciRS-7), is the most well characterized circRNA with miRNA sponge function. It contains more than 70 selectively conserved miRNA target sites and associates with Argonaute (AGO) proteins in a miR-7-dependent manner [34]. Overexpression of ciRS-7 leads to a significant decrease in miR-7 activity and results in increasing the miR-7 target gene expression level [34]. CiRS-7 is highly expressed in mammalian brain during neuronal development [29]. Studies in mice have revealed an overlapping coexpression of ciRS-7 and miR-7 in brain tissues, which indicates that this circRNA may be crucial for normal neuronal development [34]. In addition to miR-7 binding sites, ciRS-7 contains an additional binding site for miR-671. The combination of ciRS-7 and miR-671 triggers the linearization and AGO2-mediated cleavage of ciRS-7, which enables the release of the absorbed miR-7 molecules [42]. Mouse testis-specific sex-determining region Y (*Sry*) linear isoform is expressed in the developing genital ridge and has a fundamental role as a transcription factor in sex determination [43]. However, in adult testes, the circular form of *Sry* is expressed, with its circularization being dictated by promotor usage and dependent on intron pairing-driven circularization [43, 44]. *Sry* circRNA serves as the miR-138 sponge and contains 16 miR-138 binding sites [34]. Furthermore, cir-ZNF609 [45], mm9_circ_012559 [46], and circRNAs from the human C_2_H_2_ zinc finger gene family [47] have also been assumed to function as miRNA sponges. Overall, when compared to linear miRNA sponges, circRNAs have proven themselves as more stable and therefore more effective [48, 49]. Also, their expression is not affected upon miRNA binding [34]. On the other hand, several studies implied that most circRNAs do not act like miRNA sponges, since the majority does not have more miRNA binding sites than colinear mRNAs [47, 50].

It has been demonstrated that circRNAs can bind to several RBPs (Figure 2(b)), including AGO [9, 34], RNA polymerase II [17], QKI [27], EIF4A3 [51], and MBL [28]. In addition, some ecircRNAs can store, sort, or localize RBPs and presumably regulate the RBP function by acting as competing elements, in a similar way as they affect miRNA activity [9, 35].

CircRNAs form a large group of transcriptional and posttranscriptional regulators, and their direct involvement in regulating gene expression has been demonstrated in several studies [9, 17, 21]. CircRNAs (e.g., EIciRNAs and ciRNAs) abundant in the nucleus displayed little target miRNA binding sites, and knockdown of these circRNAs commonly resulted in reduced expression of their parental genes [17, 21]. EIciRNAs, such as circEIF3J and circPAIP2, interact with U1 small nuclear ribonucleoprotein (snRNP) and RNA polymerase II and upregulate their parental genes in *cis* [21] (Figure 2(e)). Thus, these findings strongly imply that circRNAs could function as scaffolds for RBPs regulating transcription, as it had been determined for several lncRNAs [52, 53]. During splicing, circRNAs and their corresponding linear isoforms may compete with each other for biogenesis [28]. However, the generated circular RNA forms may promote both circRNA and mRNA expression [21]. In addition, some circRNAs may also regulate protein expression by sequestering mRNA translation start sites [22] (Figure 2(c)).

It was shown that peptides could be translated from ecircRNAs both *in vitro* and *in vivo*, when RNA molecules contained the internal ribosomal entry site (IRES) elements or prokaryotic ribosome binding sites [36, 54, 55] (Figure 2(d)). A recent study has strongly supported the fact that a subset of *Drosophila* endogenous circRNAs has the ability to be translated *in vivo* in a cap-independent manner. Many of these translating ribosome-associated circRNAs (ribo-circRNAs) shared the start codon with the hosting gene, had evolutionary conserved stop codons, and encoded at least one identifiable ribo-circRNA-specific protein domain, implying these proteins are functional [37]. Another endogenous protein-coding circRNA, circ-ZNF609, has been identified in murine and human myoblasts. Circ-ZNF609 is involved in regulating myoblast proliferation and is generated from the second exon of its host gene. Its open reading frame (ORF) contains a start codon, common with the linear transcript, and an in-frame stop codon, created upon circularization. Circ-ZNF609 is translated into a protein in a splicing-dependent and cap-independent manner. However, molecular activity of circ-ZNF609-derived proteins still needs to be determined [38]. It was also shown that the *N*^6^-methyladenosine (m^6^A) RNA modification promotes endogenous circRNA translation in human cells. The study revealed that m^6^A motifs are enriched in many circRNAs and enable protein translation in a cap-independent manner, involving the m^6^A reader YTHDF3 and translation initiation factors eIF4G2 and eIF3A [39]. In addition, association of many circRNAs with polysomes has been determined [37–39]. These emerging insights into circRNA protein-coding ability and their further characterization may eventually reveal a vast assortment of thus far uncharacterized proteins and throw light on the processes they are involved in. Thus, it is getting progressively clearer that beside their initially proposed regulatory role as ncRNAs, circRNAs may also represent a novel type of protein-coding RNA.

## 4. CircRNA Databases and Detection Tools

### 4.1. CircRNA Databases

There are several existing circRNA databases that summarize and integrate the data obtained from large-scale circRNA identification studies, utilizing next-generation sequencing technology, which were performed by different research groups. These databases enable a transparent and comprehensive view on the spatiotemporal presence and function of circRNAs in various biological processes in different organisms, tissues, and cell types. The 9 most acknowledged circRNA databases, containing information on human and animal circRNAs, are presented in Table 1, along with their main features.

The circ2Traits database is a compiled collection of data on circRNAs, categorized according to their potential association with specific human diseases. CircRNAs are grouped based on the number of disease-associated SNPs, AGO interaction sites, and their potential interaction with disease-associated miRNAs, as determined by genome-wide association studies. circ2Traits also stores complete putative miRNA-circRNA-mRNA-lncRNA interaction networks for each of the described diseases [56].

The circBase database provides merged and unified datasets of circRNAs and the evidence supporting their expression. It is a database repository, containing circRNA data from various samples of several species, including human, mouse, fruit fly, and nematode. circBase enables exploring of public circRNA datasets and also provides custom python scripts, needed to discover novel circRNA from user's own (RiboMinus) RNA-seq data. All circRNA transcripts deposited in circBase have been annotated, their putative splice forms predicted and, where applicable, alignments of reads spanning head-to-tail junctions provided [57].

CircInteractome is a web tool designed for mapping RBP and miRNA binding sites on human circRNAs. CircInteractome also enables the identification of potential circRNAs that act as RBP sponges, designing junction-spanning primers for specific circRNA detection, designing siRNAs for circRNA silencing, and identifying potential IRES [51]. However, CircInteractome displays limited ability to predict RBP and miRNA interactions when circRNAs form secondary or tertiary structures. Thus, experimental validation is often needed to reliably verify RBP and miRNA functional sites [51].

The CircNet database provides information on novel circRNAs, integrated miRNA target networks, expression profile of circRNA isoforms, genomic annotation of circRNA isoforms, and sequence features of circRNA isoforms. CircNet also provides tissue-specific circRNA expression profiles, circRNA-miRNA-gene regulatory networks, and a thorough expression analysis of previously reported and novel circRNAs. Furthermore, CircNet generates an integrated regulatory network that illustrates the regulation between circRNAs, miRNAs, and genes [58].

CIRCpedia is an integrative database, which employs the CIRCexplorer2 characterization pipeline for identifying and annotating back-splicing and alternative splicing in circRNAs across different cell lines. Identified back-splicing and alternative splicing in circRNAs, together with novel exons, are formatted and classified for being easily searched, browsed, and downloaded. Currently, CIRCpedia contains information on circRNAs from human, mouse, fly, and worm samples [19].

The circRNADb database is a comprehensive database for human circRNAs with protein-coding annotations. circRNADb provides detailed information on circRNA genomic properties, exon splicing, genome sequences, annotated protein-coding potential, IRES, ORF, and corresponding references [59].

The starBase v2.0 database has been developed to systematically identify RNA-RNA and protein-RNA interaction networks from large-scale CLIP-seq datasets, generated by independent studies. starBase v2.0 distinctive features facilitate annotation, graphic visualization, analysis, and discovery of miRNA-mRNA, miRNA-circRNA, miRNA-pseudogene, miRNA-lncRNA, and protein-RNA interaction networks and RBP binding sites [60].

deepBase v2.0 is a platform for annotating and discovering small ncRNAs, lncRNAs, and circRNAs from next-generation sequencing data. deepBase v2.0 provides a set of tools to decode evolution, spatiotemporal expression patterns, and functions of diverse ncRNAs across 19 species from 5 clades, including human, mouse, fruit fly, and nematode. The platform also provides an integrative, interactive, and versatile web graphical interface to display multidimensional data and facilitates transcriptomic research and the discovery of novel ncRNAs [61].

TSCD (Tissue-Specific CircRNA Database) is an integrated database designed for depositing features of human and mouse tissue-specific circRNAs. TSCD provides a global view on tissue-specific circRNAs and holds information on their genomic location and conservation [62].

### 4.2. CircRNA Detection Tools

The detection and integration of newly discovered circRNAs into circRNA databases are predominantly dependent on complex bioinformatics analyses of large-scale RNA-seq data. In order to identify and annotate novel circRNAs, RNA-seq data undergoes thorough and rigorous analysis utilizing various state-of-the-art circRNA detection tools and software packages. A comprehensive overview and evaluation of 11 different circRNA detection computational pipelines have been summarized in a recent review by Zeng et al. [63]. Among the described circRNA detection tools, which were also compared with regard to their precision and sensitivity, were circRNA_finder [64], CIRCexplorer [24], DCC [65], find_circ [9], UROBORUS [66], PTESFinder [67], KNIFE [68], CIRI [69], MapSplice [70], segemehl [71], and NCLscan [72]. In general, these circRNA detection tools can be divided into two categories, based on the different strategies used for circRNA identification, according to the dependency on genome annotation [63]. In the pseudo-reference-based [73] or candidate-based approach [22], all possible combinations of candidate circRNAs are constructed. Each candidate comprises two well-annotated exons in which the exon order is topologically inconsistent with the reference genome. The candidate is regarded as a circRNA if at least one RNA-seq read has been identified, which maps to its noncolinear junction site. The strategy relies on candidate circRNAs that are constructed from preexisting gene models and does not detect circRNAs from unannotated transcripts [22, 73]. Conversely, the fragmented-based [73] or segmented read approach [22] does not rely on genome annotation. In this approach, RNA-seq reads are mapped to genomic locations de novo. Reads that cannot be mapped directly are split into two or more segments, and each segment is mapped separately to the reference genome. CircRNA back-splicing junctions are identified, when segmented reads are mapped in a noncolinear manner [22, 73]. As described by Zeng et al. [63], the tools that use the pseudo-reference-based approach to detect circRNAs include PTESFinder, KNIFE, and NCLscan, whereas circRNA_finder, CIRCexplorer, DCC, find_circ, UROBORUS, CIRI, MapSplice, and segemehl utilize the fragmented-based approach. In addition, several integrated tools aimed to identify circRNAs with a protein-coding potential have been developed, including CircPro [74], IRESite [75], CPAT [76], Pfam 31.0 [77], PhyloCSF [78], and ORF Finder from the NCBI database. Also, the TopHat-Fusion algorithm has been designed to detect circRNAs that are derived from gene fusion events [79].

Despite several useful state-of-the-art circRNA detection tools exist, their regular upgrades and the introduction of novel, improved methods, with even higher precision and sensitivity, are a prerequisite to overcome current and future challenges in circRNA identification and characterization studies.

## 5. CircRNAs in Cancer

Current knowledge about the involvement of circRNAs in cancer development and progression is limited, and the role of circRNAs as miRNA sponges has been proposed as the most frequent mechanism of circRNA activity in tumor cells [80]. Generally, miRNAs are included in various cell processes, including cellular differentiation, development, proliferation, and apoptosis, where they play an important role as regulators of gene expression [81]. These miRNA-mediated processes are frequently deregulated in cancer and can contribute to cancer initiation and progression [81]. Since many circRNAs regulate miRNA action through sponge-like binding (several are presented in Table 2), dysregulation in circRNA expression may affect their interaction with tumor-associated miRNAs, indicating an important role of circRNAs in regulating cancer.

There is emerging evidence that miR-7 can directly downregulate oncogenes in a variety of cancers [82, 83]. miR-7 has been shown to be involved in suppressing melanoma [84], breast cancer [85], glioma [86], gastric cancer [87], liver cancer [88], non-small-cell lung cancer (NSCLC) [89], colorectal cancer [90], and other cancer types. Since circRNA ciRS-7 acts as a miR-7 sponge, quenching the activity of miR-7 may increase the expression levels of miR-7 target oncogenes, resulting in a decreased tumor suppression [83]. Thereby, the ciRS-7/miR-7 axis is likely involved in cancer-related pathways and cancer development and progression [83]. However, despite its potential oncogenic properties, only few studies have revealed ciRS-7 involvement in cancer development through miR-7 binding. CiRS-7-mediated oncogenic activity, acting partly through targeting miR-7, was recently demonstrated in cancer tissues of hepatocellular carcinoma (HCC) patients. When compared to healthy adjacent tissues, ciRS-7 expression levels were significantly upregulated and ciRS-7 expression inversely correlated with miR-7 [91]. In addition, knockdown of ciRS-7 in HCC cell lines suppressed cell invasion and proliferation through miR-7 targeting [91]. Similarly, elevated expression of ciRS-7 was determined in colorectal cancer tissues, when compared to those of the adjacent normal mucosa, which was positively associated with tumor size, T stage, lymph node metastasis, and poor overall survival [92]. Downregulation of ciRS-7 increased miR-7 expression and significantly suppressed colorectal cancer cell proliferation and invasion. As demonstrated, ciRS-7 blocked miR-7 activity and positively regulated the expression of *EGFR* and *IGF-1R* oncogenes, indicating that the ciRS-7/miR-7 axis was associated with colorectal cancer progression. However, ciRS-7 may also regulate colorectal cancer progression through other mechanisms than as a miR-7 sponge [92]. Conversely, miR-7 overexpression has also been associated with upregulated oncogenes in several tumor cell lines and advanced colorectal cancer tissues, when compared to healthy controls [93]. This suggests that miR-671-mediated degradation of ciRS-7 may diminish the ciRS-7-mediated miR-7 inhibition and enhance miR-7 levels in tumor cells [83]. As a consequence, such miR-671 action may contribute to the increase in downstream target oncogenes (e.g., *EGFR* and *XIAP*) and promote vascularization, metastasis, and amplification of tumor cells [83]. It was also demonstrated that overexpression of miR-671-5p in glioblastoma multiforme (GBM) biopsies and cell lines increased the migration and proliferation rates of GBM cells [94]. Furthermore, overexpression of miR-671-5p negatively correlated with the expression of ciRS-7, *CDR1*, and *VSNL1*, which implied that the miR-671-5p/CDR1as/CDR1/VSNL1 axis was functionally altered in GBM [94].

The tumor suppressor gene *FOXO3* encodes two ncRNAs, the pseudogene Foxo3P and circ-Foxo3, both of which may act as miRNA sponges. Foxo3P and circ-Foxo3 were highly expressed in noncancerous cells and could function as miRNA sponges for several cancer-associated miRNAs, including miR-22, miR-136, miR-138, miR-149, miR-433, miR-762, miR-3614-5p, and miR-3622b-5p. However, among the two, circ-Foxo3 appeared to possess a stronger sponging effect on these miRNAs [95]. In human breast cancer cell lines, Foxo3P and circ-Foxo3 promoted the translation of Foxo3 mRNA by binding regulatory miRNAs and increased Foxo3-mediated apoptosis. Also, mouse xenograft models for breast cancer showed arrested tumor growth in the presence of circ-Foxo3 due to apoptosis, induced through combining circ-Foxo3, Foxo3P, and Foxo3 activity, when compared to controls [95]. Ectopic expression of circ-Foxo3 can result in the formation of the circ-Foxo3-p21-CDK2 ternary complex, arising from binding circ-Foxo3 to cell cycle proteins CDK2 and p21. The circ-Foxo3-p21-CDK2 ternary complex can suppress cell cycle progression and inhibit tumor growth [96]. In addition, expression of circ-Foxo3 was significantly increased during breast cancer cell apoptosis, where circ-Foxo3 effectively bound to p53 and MDM2 proteins. As demonstrated, elevated expression of circ-Foxo3 increased Foxo3 protein levels but repressed p53 activity by promoting MDM2-induced p53 ubiquitination and subsequent degradation. Overexpression of circ-Foxo3 decreased the interaction between Foxo3 and MDM2, increased Foxo3 activity, and promoted cell apoptosis, through upregulating Puma expression [97]. Beside circ-Foxo3, circ-ABCB10 is another circRNA associated with breast cancer. Circ-ABCB10 was significantly upregulated in breast cancer tissues, and its function as a sponge for miR-1271 has been determined. Furthermore, *in vitro* circ-ABCB10 knockdown suppressed proliferation and increased apoptosis of breast cancer cells [98].

Hsa_circ_001569 was significantly overexpressed in colorectal cancer tissues and was positively correlated with the degree of clinical features (TNM stage) [99]. Hsa_circ_001569 may act as a miR-145 sponge and represses the transcriptional activities of miR-145, enabling upregulation of miR-145 target genes *E2F5*, *BAG4*, and *FMNL2*. Thus, it acts as a positive regulator in cell proliferation and invasion of colorectal cancer [99]. Elevated expression levels of hsa_circ_001569 were also determined in HCC tissues, when compared to adjacent normal tissues. As in colorectal cancer, expression of hsa_circ_001569 correlated with tumor differentiation and TNM stages in HCC. The inhibitory effect on HCC cell proliferation and tumor growth through hsa_circ_001569 silencing was also demonstrated [100].

Among many dysregulated circRNAs in several cancer types, a significant upregulation of circHIPK3 in HCC has been demonstrated, when compared with its expression in matched normal tissues [101]. CircHIPK3 could bind to 9 miRNAs with its 18 potential binding sites, including the tumor-suppressive miR-124, thus inhibiting its activity. Furthermore, circHIPK3 silencing significantly inhibited human cancer cell proliferation [101]. By inhibiting miR-124 activity, circHIPK3 might influence the proliferation of tumor cells in prostate cancer through several oncogenes, including *iASPP* [102]. CircHIPK3 was also significantly downregulated in bladder cancer tissues and cell lines, where it negatively correlated with cancer grade, invasion, and lymph node metastasis [103]. Mechanistic studies revealed that circHIPK3 abundantly sponged miR-558 and suppressed heparanase (HPSE) expression, which is involved in regulating tumor invasion and metastasis. In addition, overexpression of circHIPK3 effectively inhibited migration, invasion, and angiogenesis of bladder cancer cells *in vitro* and suppressed bladder cancer growth and metastasis *in vivo*, mainly through targeting the miR-558/heparanase axis [103].

CircRNA cir-ITCH may act as a miRNA sponge for cancer-associated miR-7, miR-17, and miR-214 in esophageal squamous cell carcinoma (ESCC) [104] and miR-7 and miR-20a in colorectal cancer [105] and as a sponge for miR-7 and miR-214 in lung cancer [106]. Cir-ITCH expression was downregulated in ESCC, colorectal, and lung cancer tissues, when compared to adjacent peritumoral tissues. In all three cancer types, cir-ITCH activity could increase the level of ITCH protein, a regulator of several tumor-associated proteins, which is involved in the inhibition of the Wnt/*β*-catenin signaling pathway. Therefore, cir-ITCH likely plays an inhibitory role in ESCC and colorectal and lung cancer, through promoting ITCH-mediated ubiquitination and subsequent proteasome-mediated degradation of phosphorylated Dvl2 scaffold protein, which impairs the canonical Wnt/*β*-catenin signaling [104–106].

Involvement of another circRNA in promoting ESCC has been recently demonstrated. Hsa_circ_0067934 was significantly overexpressed in ESCC tissues, when compared to adjacent healthy tissues, and its expression positively correlated with tumor differentiation, T stage, and TNM stage [107]. *In vitro* studies revealed that hsa_circ_0067934 promoted ESCC cell proliferation, and its presence in the cytoplasm suggested that hsa_circ_0067934 was involved in posttranscriptional regulation of the ESCC cell cycle [107]. However, the exact molecular function of hsa_circ_0067934 still needs to be determined. Nevertheless, it would be interesting to assess the possibility of correlation between hsa_circ_0067934 and cir-ITCH in the development of ESCC, due to their apparent contrary modes of action.

Identification of four circRNAs associated with lung cancer has been performed, based on computational predictions utilizing transcriptome sequencing datasets, by using the CircNet database. As demonstrated, circRNAs circ-ZEB1.5, circ-ZEB1.19, circ-ZEB1.17, and circ-ZEB1.33 were upregulated in normal lung tissues, when compared to lung cancer samples, and are presumably implicated in lung cancer suppression by binding to miR-200a-3p [58], which has been reported to target *ZEB1* and to promote cancer initiation [108]. Similarly, bioinformatics approaches utilizing correlated coexpression networks of bladder cancer revealed a probable interaction between lncRNA H19 and circRNA circMYLK, demonstrating their ability to competitively bind to miR-29a-3p. Such miR-29a-3p targeting might increase the expression of *DNMT3B*, *VEGFA*, and *ITGB1* oncogenes, which suggested a possible involvement of H19 and circMYLK in the development, growth, and metastasis of bladder cancer [109].

CircRNA_100290 was upregulated and coexpressed with CDK6, a member of the cyclin-dependent kinase family, in oral squamous cell carcinoma (OSCC) tissues, when matched with noncancerous tissue samples [110]. CircRNA_100290 could directly bind to miR-29 family members, including miR-29a, miR-29b, and miR-29c. Since CDK6 has been determined as the direct target of miR-29b, circRNA_100290 evidently regulates CDK6 expression through sponging miR-29. Furthermore, knockdown of circRNA_100290 decreased the expression of CDK6, induced G1/S arrest, inhibited proliferation of OSCC cell lines *in vitro*, and decreased the growth of tumors *in vivo*. Thus, circRNA_100290 likely functions as a regulator of cell cycle and cell proliferation [110].

Involvement of two circRNAs in regulating osteosarcoma has been demonstrated recently. However, no correlation between the two in promoting osteosarcoma has been determined yet. Hsa_circ_0016347 was significantly upregulated in osteosarcoma tissues and cell lines, when compared to adjacent nontumor tissues and normal osteoblasts, and has been shown to sponge miR-214 [111], which is a known tumor promoter in osteosarcoma [112, 113]. By inhibiting miR-214 activity, hsa_circ_0016347 increased the expression level of caspase-1, a direct target of miR-214, thus enabling the formation of favorable tumor microenvironment and promoting proliferation, invasion, and metastasis of osteosarcoma cells [111]. Also, overexpression of hsa_circ_0016347 increased either the size or the number of pulmonary metastasis tumors [111]. In addition to hsa_circ_0016347, a significant upregulation of hsa_circ_0001564 has been determined in osteosarcoma tissues and cell lines, which acted as a miR-29c-3p sponge [114]. Through inhibiting miR-29c-3p activity, hsa_circ_0001564 promoted tumorigenesis of osteosarcoma by regulating cell cycle and proliferation of osteosarcoma cells [114].

As demonstrated before, circRNA_100269 has been included in a group of circRNAs constituting the four-circRNA-based classifier, which was used to predict the early recurrence of stage III gastric cancer after radical surgery [115]. Further analysis has revealed a significantly downregulated level of circRNA_100269 in gastric cancer tissues, than in the corresponding adjacent healthy tissues, which correlated with histological subtypes and the node invasion number [116]. The study suggested that circRNA_100269 inhibits gastric cancer cell proliferation via inhibiting miR-630 activity, whose expression was negatively correlated with that of circRNA_100269 [116]. However, no confirmation of such circRNA_100269 action has been performed *in vivo*.

A negative correlation between expression profiles of miR-138 and hsa_circ_0020397 was determined in colorectal cancer cells, where hsa_circ_0020397 was significantly upregulated [117]. As determined, hsa_circ_0020397 acted as a miR-138 sponge and promoted the expression of miR-138 targets TERT and PD-L1, which promoted viability and invasion of colorectal cancer cells and inhibited their apoptosis [117]. In addition, circRNA hsa_circ_0000069 was also associated with colorectal cancer and was significantly upregulated in colorectal cancer tissues and cell lines, when compared to healthy controls [118]. Elevated expression of hsa_circ_0000069 correlated with the tumor TNM stage and could promote colorectal cancer cell proliferation, invasion, and migration *in vitro* [118]. However, a more detailed mechanism of hsa_circ_0000069 function still needs to be determined.

miR-217 is a tumor-suppressive miRNA, associated with various cancer types, including epithelial ovarian cancer [119] and gastric cancer [120]. In glioma, miR-217 negatively correlated with the pathological grades of tumors and exerted tumor-suppressive activity in glioma cells [121]. CircRNA circ-TTBK2 was significantly upregulated in glioma tissues and cell lines and acted as a miR-217 sponge. By sequestering miR-217 activity, circ-TTBK2 enabled higher expression of oncogenic proteins HNF1*β* and Derlin-1, which promoted cell proliferation, migration, and invasion, while inhibiting apoptosis of glioma cells [121]. As demonstrated, miR-217 expression was negatively regulated by circ-TTBK2 expression in an AGO2-dependent manner and there was a reciprocal repression feedback loop between circ-TTBK2 and miR-217 [121]. In addition, circ-TTBK2 knockdown in combination with miR-217 overexpression led to tumor regression *in vivo* [121].

In addition to circRNAs that predominantly act like miRNA sponges, few circRNAs appear not to function in such a manner (Table 2). It has been demonstrated that downregulation of cZNF292 suppresses human glioma tube formation via the Wnt/*β*-catenin signaling pathway. Thus, cZNF292 downregulation also resulted in inhibition of glioma cell proliferation and cell cycle progression [122]. However, the exact mechanism of cZNF292 activity still needs to be determined. Furthermore, chromosomal translocations may give rise to oncogenic fusion proteins that are often involved in the onset and progression of various cancers. Such cancer-associated chromosomal translocations may also result in the formation of fusion circRNAs (f-circRNAs), which are produced from transcribed exons of genes affected by these oncogenic translocations [123]. Among several distinctive chromosomal translocations in leukemia, *PML/RARα* is the most frequent translocation in acute promyelocytic leukemia (APL) [124], which can generate one or more f-circRNAs from this fusion gene [123]. In addition, *MLL/AF9* aberrant translocation also generated several f-circRNAs in APL [123]. As demonstrated, f-circRNAs in combination with other oncogenic stimuli, including oncogenic fusion proteins, played an important role in promoting APL cell proliferation, transformation, and tumorigenesis progression *in vivo* [123]. In addition to APL, f-circRNAs have also been identified in SK-NEP-1 sarcoma and H3122 lung cancer cell lines [123]. Thus, this study strongly implied that f-circRNAs may have a potential diagnostic and therapeutic value in cancer.

In addition to the above-listed circRNAs, several research groups have identified a vast assortment of circRNAs, by using RNA-seq and other next-generation sequencing techniques that are likely involved in mechanisms which promote various cancers. The majority of the generated data can be obtained from several circRNA databases, which are presented in Table 1. Despite candidate cancer-specific circRNAs are getting discovered on a regular basis, the data currently remains insufficient to definitely associate individual circRNAs with a specific mechanism promoting a certain cancer type. However, it has recently become clear that circRNAs may represent promising biomarkers for various cancer types.

## 6. CircRNAs as Cancer Biomarkers

CircRNAs are abundant and highly stable molecules, exhibiting high cell/tissue and developmental stage specificity [9, 11]. The unique circular structure makes circRNAs insensitive to ribonucleases and enables them to exist intact in various tissues and body fluids. It has been shown that circRNAs may be stably expressed and present in relatively high quantities in human blood [125], saliva [126], and exosomes [33]. These characteristics make circRNAs ideal candidates as noninvasive biomarkers for cancer diagnosis, prognosis, and treatment. In addition, some circRNAs may correlate with age, gender, TNM stage, metastasis, and tumor size as it was determined for gastric cancer [16], HCC [127, 128], and colorectal cancer [99], additionally implying their suitability as cancer biomarkers. A number of circRNAs that have been associated with human cancer are presented in Table 2. From these circRNAs, several have been tested for their diagnostic performance and may eventually become novel biomarkers for cancer diagnosis in clinical practice.

### 6.1. CircRNAs as Biomarkers for Bladder Cancer

CircRNA circTCF25 was found to be highly expressed in bladder cancer tissues, when compared to healthy controls. The analysis was performed by using a total of 40 paired snap-frozen bladder carcinoma and matched paracarcinoma tissue samples. The study showed that circTCF25 promotes proliferation and metastasis of urinary bladder carcinoma by acting as a sponge for miR-103a-3p and miR-107, which resulted in upregulated CDK6 expression [129]. The data also suggested that circTCF25 may be a new promising biomarker for bladder cancer [129]. However, the diagnostic performance of this circRNA still needs to be determined.

### 6.2. CircRNAs as Biomarkers for Colorectal Cancer

CircRNA hsa_circ_001988 has been identified in colorectal cancer and has been significantly downregulated in colorectal cancer tissues, when compared to those of the matched normal mucosa (*n* = 31) [130]. Evaluation of the diagnostic performance of hsa_circ_001988 has shown its sensitivity of 68.0% and specificity of 73.0%. The receiver operating characteristic curve (ROC) analysis showed an area under the ROC curve (AUC) of 0.788, indicating that hsa_circ_001988 may become a novel potential biomarker in the diagnosis of colorectal cancer [130].

CircRNAs hsa_circRNA_103809 and hsa_circRNA_104700 were also significantly downregulated in colorectal cancer tissues, where hsa_circRNA_103809 correlated with lymph node metastasis and TNM stage and hsa_circRNA_104700 with distal metastasis [131]. Analysis of both circRNAs was performed on 170 paired colorectal cancer tissues and matched adjacent noncancerous tissue samples. The evaluated diagnostic performances for hsa_circRNA_103809 (AUC 0.699) and hsa_circRNA_104700 (AUC 0.616) indicated that both circRNAs may serve as reliable biomarkers for colorectal cancer [131]. However, beside their dysregulation and putative miRNA binding site determination [131], the exact mechanisms of function for both circRNAs in colorectal cancer development have not been elucidated yet.

Despite its assumed involvement in promoting various cancer types, mainly due to its ability to sponge miR-7, the clinical significance of ciRS-7 in colorectal cancer was only recently demonstrated. CiRS-7 was significantly upregulated in tumor tissues of colorectal cancer patients and correlated with advanced tumor stage, tumor depth, and metastasis [132]. The study included a training cohort comprised of 153 primary colorectal cancer tissues and 44 matched normal mucosa tissues and an additional independent validation cohort (*n* = 165). Correlation of upregulated ciRS-7 expression levels with poor patient survival strongly suggested that ciRS-7 might serve as a novel prognostic biomarker in colorectal cancer patients [132]. *In vitro* experiments revealed that ciRS-7 inhibited miR-7 activity and activated the EGFR/RAF1/MAPK pathway, which linked ciRS-7 activity with colorectal cancer progression and aggressiveness [132]. Regarding the data obtained from the study, ciRS-7 suppression could increase the expression levels of miR-7 and reduce EGFR-RAF1 activity. Thus, therapeutic targeting of ciRS-7 might represent a potential treatment option for patients with colorectal cancer [132].

In addition, elevated expression levels of circRNA circ-KLDHC10 in serum samples of colorectal cancer patients were determined, when compared to those in healthy controls (*n* = 11 for both sample groups). Since circ-KLDHC10 was abundant in exosomes, it has the potential to serve as a novel circulating biomarker for colorectal cancer [33]. However, its oncogenic activity and diagnostic performance in colorectal cancer still need to be determined.

### 6.3. CircRNAs as Biomarkers for Non-Small-Cell Lung Cancer (NSCLC)

CircRNA_100876 was significantly upregulated in NSCLC tissues, when compared to their paired adjacent nontumorous tissues (*n* = 101), and its elevated levels closely correlated with lymph node metastasis and advanced tumor staging in NSCLC [133]. As determined in a previous study, circRNA_100876 could regulate MMP-13 expression through inhibiting miR-136 activity and thus participated in chondrocyte extracellular matrix degradation [134]. Since MMP-13 is often overexpressed in lung cancer and can increase the risk of metastasis [135, 136], circRNA_100876 might be involved in tumor cell growth, progression, and metastasis in NSCLC, by regulating MMP-13 expression as a miRNA sponge [133]. The Kaplan-Meier survival analysis showed significantly shorter overall survival times of NSCLC patients with elevated circRNA_100876 expression levels, when compared to patients with low expression levels of circRNA_100876. Therefore, circRNA_100876 could be gradually used as a novel prognostic biomarker for NSCLC [133].

### 6.4. CircRNAs as Biomarkers for Hepatocellular Carcinoma (HCC)

Hsa_circ_0001649 was significantly downregulated in HCC tissues, when compared to paired adjacent healthy liver tissues (*n* = 89), and its expression levels correlated with tumor size and the occurrence of tumor embolus in HCC [127]. Hsa_circ_0001649 may play a role in tumorigenesis and metastasis of HCC through sponge-like activity toward several miRNAs, including miR-1283, miR-4310, miR-182-3p, miR-888-3p, miR-4502, miR-6811, miR-6511b-5p, and miR-1972 [127]. The evaluated diagnostic performance (sensitivity 81.0%; specificity 69.0%; and AUC 0.63) indicated that hsa_circ_0001649 might serve as a novel potential biomarker for HCC, with relatively high degrees of accuracy, specificity, and sensitivity [127].

Another circRNA associated with HCC is hsa_circ_0005075, which was significantly upregulated in HCC tissues, when compared to paired adjacent normal liver tissues (*n* = 30) [128]. Hsa_circ_0005075 correlated with tumor size and showed a great diagnostic potential with a sensitivity of 83.3%, specificity of 90.0%, and AUC of 0.94 [128]. In addition, the circRNA-miRNA-mRNA interaction network revealed that hsa_circ_0005075 could potentially interact with miR-23b-5p, miR-93-3p, miR-581, and miR-23a-5p. The study assumed that through its miRNA sponge-like activity, hsa_circ_0005075 may participate in regulating cell adhesion during HCC development, which is involved in cancer cell proliferation, invasion, and metastasis [128].

The relationship between ciRS-7 and clinical features of HCC was also demonstrated. In the study, ciRS-7 expression was upregulated in 39.8% (*n* = 43) and downregulated in 60.2% (*n* = 65) tissues of HCC patients, when compared to matched nontumor tissues (*n* = 108) [137]. Even though ciRS-7 expression was slightly higher in HCC tissues, the overall ciRS-7 expression levels were downregulated and not significantly different from those in healthy controls [137], which was in contrast with a previous study describing ciRS-7 involvement in HCC [91]. However, upregulated ciRS-7 expression significantly correlated with patient age, serum AFP levels, and hepatic microvascular invasion (MVI), which suggested ciRS-7 expression may be associated with deterioration and metastasis of HCC [137]. Also, ciRS-7 could promote MVI by inhibiting miR-7 and disrupting the PIK3CD/p70S6K/mTOR pathway [137]. The ROC curve analysis showed that ciRS-7 was related to MVI in HCC tissues with an AUC of 0.68, implying ciRS-7 expression level could predict MVI. Considering these results, the study indicated ciRS-7 may not be a key factor in HCC tumorigenesis, but rather a risk factor for MVI in HCC [137].

The zinc finger family gene *ZKSCAN1* can generate linear *ZKSCAN1* mRNA and circular circZKSCAN1 isoforms, both of which were associated with different regulatory roles in the development of HCC, mostly through inhibiting growth, migration, and invasion of HCC cells [138]. CircZKSCAN1 was significantly downregulated in HCC tissues, when compared to paired adjacent healthy tissues (*n* = 102), and its expression levels correlated with tumor numbers, cirrhosis, vascular invasion, MVI, and tumor grade [138]. The ROC analysis showed the AUC of circZKSCAN1 was 0.834 with a sensitivity of 82.2% and specificity of 72.4%, indicating circZKSCAN1 could be used as a biomarker to effectively differentiate cancerous tissues from adjacent noncancerous tissues in HCC [138].

In addition, two relatively recently identified tumor-suppressive circRNAs were associated with clinical characteristic of patients with HCC. Low expression levels of hsa_circ_0005986 correlated with chronic hepatitis B family history, tumor diameters, MIV, and Barcelona Clinic Liver Cancer staging system (BCLC) stage [139]. The analysis was performed on 81 paired HCC and matched nontumorous tissue samples. As determined, hsa_circ_0005986 regulated the HCC cell cycle and proliferation, by acting as a miR-129-5p sponge and through promoting *Notch1* gene expression [139]. However, despite the study suggested hsa_circ_0005986 could be used as a novel HCC biomarker, no diagnostic performance of this circRNA has been performed. Similar to hsa_circ_0005986, the decreased expression levels of hsa_circ_0004018 in HCC tissues correlated with AFP level, tumor diameters, differentiation, BCLC stage, and TNM stage, when compared to those in paired para-tumorous tissues (*n* = 102) [140]. miRNA target prediction analysis revealed that hsa_circ_0004018 could sponge miR-30e-5p, miR-647, miR-92a-5p, miR-660-3p, and miR-626, additionally implying its role in tumorigenesis of HCC [140]. The evaluated diagnostic performance (sensitivity 0.716; specificity 0.815; and AUC 0.848) along with its HCC stage-specific expression profile highlighted hsa_circ_0004018 as a suitable biomarker for HCC diagnosis, capable of distinguishing HCC tissues from healthy and chronic hepatitis tissues [140].

### 6.5. CircRNAs as Biomarkers for Gastric Cancer

Significantly downregulated expression profiles of hsa_circ_002059 have been determined in gastric cancer tissues, when compared to paired adjacent nontumor tissues (*n* = 101) [16]. In addition, hsa_circ_002059 levels in plasma were significantly different between 36 paired plasma samples from pre- and postoperative gastric cancer patients. Also, lower expression levels of hsa_circ_002059 were significantly correlated with a patient's distal metastasis, TNM stage, gender, and age. Evaluated diagnostic performance of hsa_circ_002059 has shown its sensitivity of 81.0%, specificity of 62.0%, and AUC of 0.73, indicating hsa_circ_002059 represents a potential stable biomarker for gastric cancer [16].

Beside its role as a potential biomarker in HCC, hsa_circ_0001649 has also been associated with diagnosis of gastric cancer. Hsa_circ_0001649 was significantly downregulated in gastric cancer tissues, when compared to their paired paracancerous histological normal tissues (*n* = 76), and its expression levels correlated with pathological differentiation [141]. Analysis of hsa_circ_0001649 serum expression levels between paired pre- and postoperative serum samples (*n* = 20) of gastric cancer patients showed that hsa_circ_0001649 was significantly upregulated in serum after surgery. Also, hsa_circ_0001649 expression levels were more significantly decreased in poor and undifferentiated tumors than in well-differentiated ones, indicating its potential negative correlation with gastric cancer pathological differentiation [141]. The estimated diagnostic value of hsa_circ_0001649 determined by the ROC analysis showed the AUC of 0.834, with a sensitivity and specificity of 0.711 and 0.816, respectively [141]. These results suggest hsa_circ_0001649 may become a novel noninvasive biomarker for early detection of primary gastric cancer.

Hsa_circ_0000096 is a tumor-suppressive circRNA that affects gastric cancer cell growth and migration through suppressing the expression levels of cell cycle-associated (cyclin D1, CDK6) and migration-associated (MMP-2, MMP-9) proteins. Furthermore, hsa_circ_0000096 may also interact with 17 types of miRNA, including miR-224 and miR-200a [142]. Hsa_circ_0000096 was significantly downregulated in gastric cancer tissues (compared to paired adjacent nontumorous tissues; *n* = 101), and cell lines and its aberrant expression correlated with invasion and TNM stage [142]. The ROC analysis showed the AUC of hsa_circ_0000096 was 0.82. Intriguingly, the AUC was increased to 0.91 when a combination of hsa_circ_0000096 and a previously described hsa_circ_002059 was used [142]. Thus, these results suggest that hsa_circ_0000096 could be used independently or in combination with hsa_circ_002059 for effective diagnosis of gastric cancer.

In addition, four circRNAs have been recently proposed as biomarkers for gastric cancer, all of which were statistically significantly downregulated in gastric cancer tissues, correlated with different clinical characteristics, and showed an excellent diagnostic potential with relatively high accuracy, specificity, and sensitivity [143–146]. Hsa_circ_0001895 expression levels were downregulated in 69.8% (*n* = 67) gastric cancer tissues, compared to paired adjacent normal tissues (*n* = 96), and significantly correlated with cell differentiation, Borrmann type, and tissue CEA expression. The evaluated diagnostic performance of hsa_circ_0001895 showed the AUC was up to 0.792 with a sensitivity and specificity of 67.8% and 85.7%, respectively. The optimal cutoff value was 9.53 [143]. Interestingly, better sensitivity and specificity were obtained with the use of hsa_circ_0001895, when compared to common gastric cancer biomarkers CEA, CA19-9, and CA72-4, which showed only 20.1–27.6% sensitivity individually or 48.2% when used in combination [147]. This indicates hsa_circ_0001895 may be effectively used for screening and predicting the prognosis of gastric cancer [143]. Downregulation of hsa_circ_0006633 was associated with cancer distal metastasis and tissue CEA levels. In the study, 96 paired gastric cancer tissues and their adjacent nontumorous tissues were used. The evaluated diagnostic performance (sensitivity 0.60; specificity 0.81; and AUC 0.741) and increased hsa_circ_0006633 levels in plasma samples suggested that this circRNA could be used as a novel noninvasive biomarker for screening gastric cancer [144]. Hsa_circ_0000190 levels in gastric cancer tissues were correlated with tumor diameter, lymphatic metastasis, distal metastasis, TNM stage, and CA19-9 levels. However, in plasma samples, hsa_circ_0000190 correlated only with CEA levels [145]. The analysis was performed by using 104 paired gastric cancer tissues and their adjacent nontumor tissues, 104 plasma samples from gastric cancer patients, and 104 plasma samples from healthy controls. The diagnostic potential of hsa_circ_0000190 was determined in tissue (sensitivity of 0.721, specificity of 0.683, and AUC of 0.75) and plasma (sensitivity of 0.414, specificity of 0.875, and AUC of 0.60) samples. When tissue and plasma hsa_circ_0000190 were combined, the AUC was increased to 0.775, with a sensitivity and specificity of 0.712 and 0.750, respectively [145]. Moreover, when compared to CEA and CA19-9, hsa_circ_0000190 had a much higher sensitivity and specificity in the screening of gastric cancer [145]. Low hsa_circ_0003159 tissue levels in gastric cancer patients correlated with gender, distal metastasis, and TNM stage. The analysis was performed on 108 paired gastric cancer tissues and adjacent nontumorous tissue samples. The determined sensitivity and specificity were 0.852 and 0.565, respectively. The AUC of hsa_circ_0003159 was 0.75, which indicated that hsa_circ_0003159 may be also used as a biomarker for the diagnosis of gastric cancer [146].

In contrast to the above-listed circRNAs, circPVT1 expression levels were significantly upregulated in gastric cancer cells and tissues, when compared to paired healthy controls (*n* = 187) [148]. As demonstrated, circPVT1 may promote gastric cancer proliferation through acting as a sponge toward the miR-125 family members [148]. The Kaplan-Meier analysis confirmed that circPVT1 could serve as an independent prognostic biomarker for the overall survival and disease-free survival of patients with gastric cancer. Furthermore, the combined detection of expressed circPVT1 and its linear isoform from the *PVT1* oncogene enhanced the prognosis of patients with gastric cancer [148]. In addition, statistically significant upregulation of hsa_circ_0058246 was detected in tumor specimens of gastric cancer patients with poor clinical outcomes (*n* = 43). Also, patients who suffered recurrence of gastric cancer (*n* = 12) had a significant increase in hsa_circ_0058246 expression levels [149]. However, further studies are needed to convincingly demonstrate the suitability of hsa_circ_0058246 in diagnosis and prognosis of gastric cancer.

### 6.6. CircRNAs as Biomarkers for Laryngeal Squamous Cell Cancer (LSCC)

Microarray and subsequent qRT-PCR analyses of laryngeal squamous cell cancer (LSCC) tissues have shown hsa_circ_100855 as the most upregulated and hsa_circ_104912 as the most downregulated circRNA in LSCC [150]. Hsa_circ_100855 levels were significantly higher in LSCC tissues and in patients with T3-4 stage, neck nodal metastasis, or advanced clinical stage. Conversely, hsa_circ_104912 levels were significantly lower in LSCC tissues than in corresponding adjacent nonneoplastic tissues. The study included 4 matched samples of LSCC tissues and corresponding adjacent nonneoplastic tissues for microarray analysis and 52 matched cancerous and noncancerous tissues for qRT-PCR analysis. Despite no diagnostic performance of either circRNAs has been determined, hsa_circ_100855 and hsa_circ_104912 may both serve as potential biomarkers and therapeutic targets for LSCC [150].

## 7. Conclusions and Perspectives

CircRNAs appear to be stably expressed in a cell/tissue-dependent and developmental stage-specific manner and have been shown to be dysregulated in different cancers. They are generally more stable than miRNAs and lncRNAs, several of which are currently recognized as relatively well-established biomarkers in cancer diagnosis. However, before circRNAs could be routinely used as effective biomarkers for early cancer diagnosis and prognosis, several important issues should be addressed, including the determination of their diagnostic performances for specific cancer types. As demonstrated above, several circRNAs have shown satisfactory diagnostic performances in distinguishing tumor from healthy tissues and between specific cancer types. However, suitable circRNAs as independent cancer biomarkers have not been identified yet. CircRNAs could be used in combination with RNA-based and other conventional cancer biomarkers, such as CEA, CA125, CA153, PSA, and AFP, for more specific cancer diagnosis and accurate cancer prognosis. In addition, their stability and abundance in exosomes suggest that circRNAs may represent a new class of exosome-based noninvasive cancer biomarkers. CircRNAs may also have a potential in targeted cancer treatment, where they could be utilized as sponges to bind to aberrantly expressed regulatory RNAs and proteins (e.g., RBPs), thus diminishing their oncogenic activity. However, to achieve this, further insights into circRNA's molecular mechanisms and functions in circRNA-mediated diseases are a prerequisite, before such treatments may become applicable. Also, identification of dysregulated circRNAs in other body fluids, such as urine and cerebrospinal fluid, may be beneficial for noninvasive cancer diagnosis. To conclude, circRNAs represent promising novel biomarkers for various cancer types and have a great potential to be effectively used in clinical practice in the near future.

## Figures and Tables

**Table 1:** Databases containing circRNA data.

Name	URL	Description	References
circ2Traits	http://gyanxet-beta.com/circdb/	A database containing information on disease-associated circRNAs and their complete putative interaction networks with miRNAs, mRNAs, and lncRNAs in specific diseases.	[56]
circBase	http://www.circbase.org/	A collection of merged and unified datasets of circRNAs, with evidence supporting their expression	[57]
CircInteractome	https://circinteractome.nia.nih.gov/	A web tool designed for predicting and mapping RBP and miRNA binding sites on reported circRNAs	[51]
CircNet	http://circnet.mbc.nctu.edu.tw/	A database providing information on known and novel circRNAs, circRNA-miRNA-gene regulatory networks, and tissue-specific circRNA expression profiles	[58]
CIRCpedia	http://www.picb.ac.cn/rnomics/circpedia/	A database holding information on identified and annotated back-splicing and alternative splicing in circRNAs from human, mouse, fly, and worm samples	[19]
circRNADb	http://reprod.njmu.edu.cn/circrnadb	A comprehensive database for human circRNAs with protein-coding annotations	[59]
starBase v2.0	http://starbase.sysu.edu.cn/	A database for decoding predicted interaction networks between lncRNAs, miRNAs, circRNAs, mRNAs, and RBPs from large-scale CLIP-seq data	[60]
deepBase v2.0	http://deepbase.sysu.edu.cn/	A platform for annotating, discovering, and characterizing small ncRNAs, lncRNAs, and circRNAs from next-generation sequencing data	[61]
TSCD	http://gb.whu.edu.cn/TSCD/	An integrated database designed for depositing features of human and mouse tissue-specific circRNAs	[62]

**Table 2:** CircRNAs associated with cancer.

CircRNA	Gene symbol	Cancer type	Expression	Fold change^a^	Function	References
CiRS-7/CDR1as^∗^	*CDR1*	HCC	Up	NA	Sponge: miR-7	[91]
		HCC^∗^	Down^b^	NA	Sponge: miR-7	[137]
		Colorectal	Up	NA	Sponge: miR-7	[92]
		Colorectal^∗^	Up	2.4	Sponge: miR-7	[132]
		GBM	Down	3.5	Target of miR-671-5p Association with cell proliferation and migration	[94]
Circ-Foxo3	*FOXO3*	Breast	Down	NA	Sponge: miR-22, miR-136, miR-138, miR-149, miR-433, miR-762, miR-3614-5p, miR-3622b-5p Association with apoptosis-related proteins Foxo3, MDM2, p53, and Puma and cell cycle proteins CDK2 and p21	[95–97]
Circ-ABCB10	*ABCB10*	Breast	Up	5.0–10.0	Sponge: miR-1271	[98]
Hsa_circ_001569	*ABCC1*	Colorectal	Up	NA	Sponge: miR-145	[99]
		HCC	Up	NA	Promoting tumor growth	[100]
CircHIPK3	*HIPK3*	HCC	Up	NA	Sponge: miR-124	[101]
		Bladder	Down	4.6	Sponge: miR-558	[103]
Cir-ITCH	*ITCH*	ESCC	Down	NA	Sponge: miR-7, miR-17, miR-214 Inhibition of the Wnt/*β*-catenin pathway	[104]
		Colorectal	Down	NA	Sponge: miR-7, miR-20a Inhibition of the Wnt/*β*-catenin pathway	[105]
		Lung	Down	NA	Sponge: miR-7, miR-214 Inhibition of the Wnt/*β*-catenin pathway	[106]
Hsa_circ_0067934	*PRKCI*	ESCC	Up	8.8	Promoting cell proliferation and migration	[107]
Circ-ZEB1.5	*ZEB1*	Lung	Down	NA	Sponge: miR-200a-3p	[58]
Circ-ZEB1.19						
Circ-ZEB1.17						
Circ-ZEB1.33						
CircMYLK	*MYLK*	Bladder	Up	NA	Sponge: miR-29a-3p	[109]
CircRNA_100290	*SLC30A7*	OSCC	Up	6.9	Sponge: miR-29 family	[110]
Hsa_circ_0016347	*KCNH1*	Osteosarcoma	Up	NA	Sponge: miR-214	[111]
Hsa_circ_0001564	*CANX*	Osteosarcoma	Up	NA	Sponge: miR-29c-3p	[114]
CircRNA_100269	*LPHN2*	Gastric	Down	NA	Sponge: miR-630	[116]
Hsa_circ_0020397	*DOCK1*	Colorectal	Up	NA	Sponge: miR-138	[117]
Hsa_circ_0000069	*STIL*	Colorectal	Up	≥1.0	Promoting cell proliferation, invasion, and migration	[118]
Circ-TTBK2	*TTBK2*	Glioma	Up	NA	Sponge: miR-217	[121]
cZNF292	*ZNF292*	Glioma	Up	NA	Promoting cell proliferation and tube formation	[122]
f-circRNA	*PML/RARα* ^c^	APL	Up	NA	Promoting cell proliferation, transformation, and tumorigenesis	[123]
CircTCF25^∗^	*TCF25*	Bladder	Up	21.4	Sponge: miR-103a-3p, miR-107	[129]
Hsa_circ_001988^∗^	*FBXW7*	Colorectal	Down	NA	ND	[130]
Hsa_circRNA_103809^∗^	*ZFR*	Colorectal	Down	3.6	Sponge: miR-511-5p, miR-130b-5p, miR-642a-5p, miR-532-3p, miR-329-5p	[131]
Hsa_circRNA_104700^∗^	*PTK2*	Colorectal	Down	4.2	Sponge: miR-141-5p, miR-500a-5p, miR-509-3p, miR-619-3p, miR-578	[131]
CircRNA_100876^∗^	*RNF121*	NSCLC	Up	1.2	Sponge: miR-136	[133]
Hsa_circ_0001649^∗^	*SHPRH*	HCC^∗^	Down	NA	Sponge: miR-1283, miR-4310, miR-182-3p, miR-888-3p, miR-4502, miR-6811, miR-6511b-5p, miR-1972 Promoting metastasis	[127]
		Gastric^∗^	Down	NA	ND	[141]
Hsa_circ_0005075^∗^	*EIF4G3*	HCC	Up	NA	Sponge: miR-23b-5p, miR-93-3p, miR-581, miR-23a-5p Promoting cell adhesion	[128]
CircZKSCAN1^∗^	*ZKSCAN1*	HCC	Down	NA	Inhibition of cellular growth, migration, and invasion	[138]
Hsa_circ_0005986	*PRDM2*	HCC	Down	2.9	Sponge: miR-129-5p	[139]
Hsa_circ_0004018^∗^	*SMYD4*	HCC	Down	NA	Sponge: miR-30e-5p, miR-647, miR-92a-5p, miR-660-3p, miR-626	[140]
Hsa_circ_002059^∗^	*KIAA0907*	Gastric	Down	NA	ND	[16]
Hsa_circ_0000096^∗^	*HIAT1*	Gastric	Down	NA	Sponge: miR-224, miR-200a Inhibition of cell growth and migration	[142]
Hsa_circ_0001895^∗^	*PRRC2B*	Gastric	Down	NA	ND	[143]
Hsa_circ_0006633^∗^	*FGGY*	Gastric	Down	NA	ND	[144]
Hsa_circ_0000190^∗^	*CNIH4*	Gastric	Down	NA	ND	[145]
Hsa_circ_0003159^∗^	*CACNA2D1*	Gastric	Down	NA	ND	[146]
CircPVT1^∗^	*PVT1*	Gastric	Up	NA	Sponge: miR-125 family Promoting cell proliferation	[148]
Hsa_circ_100855^∗^	*C11orf80*	LSCC	Up	10.5	ND	[150]
Hsa_circ_104912^∗^	*DENND1A*	LSCC	Down	4.7	ND	[150]

HCC: hepatocellular carcinoma; GBM: glioblastoma multiforme; ESCC: esophageal squamous cell carcinoma; OSCC: oral squamous cell carcinoma; APL: acute promyelocytic leukemia; NSCLC: non-small-cell lung cancer; LSCC: laryngeal squamous cell cancer. Up: upregulated; down: downregulated. NA: not available (data is presented in a graphical format in the original report). ND: not determined. ^∗^Potential cancer biomarker. ^a^Fold change values, relative to normal controls. ^b^Expression levels were not statistically significant. ^c^One or more f-circRNAs were generated from PML/RAR*α* fusion gene, a product of the most recurrent cancer-associated aberrant chromosomal translocation in APL. In addition, other chromosomal translocations may also generate f-circRNAs.
